# Magnitude of surgical site infection and its associated factors among patients who underwent a surgical procedure at Wolaita Sodo University Teaching and Referral Hospital, South Ethiopia

**DOI:** 10.1371/journal.pone.0226140

**Published:** 2019-12-05

**Authors:** Nefsu Awoke, Aseb Arba, Abiy Girma

**Affiliations:** 1 Department of Nursing, College of Health Science and Medicine, Wolaita Sodo University, Wolaita Sodo, Ethiopia; 2 Wolaita Sodo University Teaching and Referral Hospital, Wolaita Sodo University, Wolaita Sodo, Ethiopia; Medical University Graz, AUSTRIA

## Abstract

**Introduction:**

Surgical site infections are infections that take place within 30 days of an operative procedure. Worldwide, 23% of patients develop surgical site infections among all surgeries annually with the worst complications causing prolonged hospital stays, increased resistance of microorganisms to antimicrobials, higher health system costs, emotional stress for patients and their families, and substantial economic burdens on hospitals. Therefore, this study was created to assess the magnitude and associated factors of surgical site infection at Wolaita Sodo University Teaching and Referral Hospital.

**Method:**

We conducted a hospital-based cross-sectional study on patients who underwent a surgical procedure in 2018 at Wolaita Sodo University Teaching and Referral Hospital. We applied a systematic random sampling technique to obtain 261 patient records from all records of surgical patients from January 1, 2018, to December 30, 2018. We collected data using a pretested checklist. We used bivariate and multivariate logistic regression analysis to identify factors associated with surgical site infection. We considered a P-value < 0.05 as statistically significant. Summary measures, texts, tables, and figures present the results of the analysis.

**Result:**

Among the 261 patients, 34 or 13% (95% CI = 9.2%, 17.2%) developed surgical site infection. Patients younger than 40 years old [AOR 6.45; 95% CI (1.56, 26.67)], illiterate [AOR 4.25; 95% CI (1.52, 11.84)], with a history of previous hospitalization [AOR 4.50; 95% CI (1.44, 14.08)], with a prolonged preoperative hospital stay (≥ 7 days) [AOR 3.88; 95% CI (1.46, 10.29)], and admitted to the public wing of the ward [AOR 0.24; 95% CI (0.07, 0.79)] possessed factors associated with surgical site infection.

**Conclusion:**

The magnitude of surgical site infection in this study was high. Shortening preoperative hospital stays, delivering intravenous antimicrobial prophylaxis before surgery, and giving wound care as ordered would significantly reduce the incidence of surgical site infection.

## Introduction

Surgical site infection (SSI) refers to infections that take place within 30 days of an operative procedure and may extend to more than 30 days according to the surgical procedure [1]. One of the common problems in a hospital setting, reports from the World Health Organization in 2009, 23% of surgical patients worldwide developed SSIs [2]. In the US, 500,000 SSIs occur every year and are the second most common health care institution infection [3]. In 2012, a São Paulo, Brazil, study revealed that 22% of 195 patients admitted to an intensive care unit developed a hospital acquired infection [4]. According to a 2012 study conducted in Nigerian pediatric hospital, 30.9% of all operation sites were infected [5].

The impact of healthcare-associated infection is multifactorial, including prolonged hospital stays, long-term disabilities, increased resistance of microorganisms to antimicrobials, high health system costs, emotional stress for patients and their families, and substantial economic burdens for hospitals. SSIs and hospital stays can lead to pressure ulcers, hypoglycemia, additional economic burden, and death [6, 7]. Different studies have shown that the most common causes of SSIs relate to inadequate supplies of personal protective equipment, a lack of training on infection control measures, an absence of hospital policy on infection control, and inadequate hand washing practices [8,9]. Infections might also be related to direct contact between a patient and an inanimate object without proper hand washing or using appropriate antisepsis [2, 6]. Excessive nursing workload is an additional factor of SSIs [4].

Most SSIs are preventable through basic and advanced nursing procedures of wound care. To provide effective infection prevention care, health care professionals should stay updated with the knowledge and skills to provide the best possible practice [5, 10]. In Sub-Saharan Africa (including Ethiopia and especially the southern part of the country), there are few evidential studies regarding the magnitude of SSI and its associated factors. Therefore, this study was created to assess the magnitude and associated factors of SSI at Wolaita Sodo University Teaching and Referral Hospital (WSUTRH).

## Method and materials

### Study setting

We conducted the study in WSUTRH. The total number of beds in the hospital is 268, covering medical, pediatrics, surgical, gynecology, and obstetrics wards. The hospital gives service to approximately 3.5–5 million patients annually. We conducted a hospital-based cross-sectional study design using a retrospective chart review. The source populations were charts of patients who underwent surgery at WSUTRH from January 1, 2018, to December 30, 2018.

### Inclusion criteria

- We included all patients who underwent surgery during the study period.

### Exclusion criteria

- We excluded patients with incomplete charts.
- We excluded patients who had undergone an operation with another institution before coming to WSUTRH for a follow-up.

### Sample size determination

We determined the sample size using a single population proportion formula and the following assumptions: p being the prevalence of 19.1% from a study conducted in Hawassa [11], d being the expected margin of error (5%), Z being the standard score corresponding to a 95% confidence interval, and α being the risk of rejecting the null hypothesis (0.05). The required sample size was determined to be 261.

### Sampling technique

A total of 3,715 patients underwent a surgical procedure at WSUTRH from January 1, 2018, to December 30, 2018. Using a systematic random sampling technique, we selected 261 patient charts at every fourteenth interval. The sampling interval was determined by dividing the total study population who underwent a surgical procedure in the last one year at WSUTRH by the sample size, and then the starting point was randomly selected by lottery method.

### Data collection tool and technique

We collected data using a pretested checklist, which we developed by reviewing different literature. Review of microbiology reports and patient medical records used indirect measurement of the surgical site infection method. The indirect method of SSI surveillance is both reliable (sensitivity, 84%–89%) and specific (specificity, 99.8%) [8, 12]. We involved two data collectors who have a BSc degree in nursing in the data collection process. Using a card number of patients, data collectors traced and collected data from randomly identified charts of a patient using a checklist.

### Data processing and analysis

We entered the collected data and analyzed the data using SPSS version 22. We assessed the statistical significance with the dependent variable at a p-value of less than 0.05. We used descriptive statistics including tables to describe the data. We performed bivariate and multivariable logistic regression analysis to see the association between dependent and independent variables. Variables that found to be statistically significant in the bivariate analysis at a p-value of less than 0.25 entered the multivariable logistic regression model. A p-value of less than 0.05 considered statistically significant in a multivariable logistic regression analysis and Odds ratio along with its 95% CI used to assess the association between dependent and independent variables. Finally, the level of statistical significance declared at a p-value less than 0.05.

### Data quality control

We did the pretest of the checklist on 5% of the sample size out of the study area to ensure its validity. Two-day training (one day theoretical and one day practical) given on the data collection tool and how to conduct data collection. The principal investigator supervised the activities of the data collector. The principal investigator checked completeness and consistency of data on a daily basis. We did double data entry by two data clerks and consistencies of the entered data were cross-checked by comparing the two separately entered data on SPSS.

### Ethical considerations

Ethical approval was first got from the Ethical Clearance Committee of Wolaita Sodo University. Then a letter of cooperation written to Wolaita Sodo University Teaching and Referral Hospital (WSUTRH) administration. Ethical Clearance Committee waived the requirement for informed consent to have data from the patient medical records. Participants’ confidentiality of information assured by excluding names and identifiers in the checklist.

## Result

### Socio-demographic characteristics

A total, 261 patients were included in the analysis. Forty-six percent of the respondents aged>40 years. Males account for a majority of 62.8% among the participants. Literate was 59.4% and 18.8% were government workers. About half of the participants were from urban in residence. The majority had a previous history of hospitalization, and 77.4% of the participants admitted in the public ward of the hospital. Sixty-seven percent of the participants have stayed in the hospital for over seven days (Table 1).

**Table 1:** Socio-demographic characteristics of study participant (n = 261).

Variables		Frequency	Percentage
Age	1–18	58	22.2
	19–40	81	31
	>40	122	46.7
Sex	Male	164	62.8
	Female	97	37.2
Educational status	Literate	155	59.4
	Illiterate	106	40.6
Occupation	Government workers	49	18.8
	Farmer	45	17.2
	Merchant	60	23
	House wife	38	14.6
	Others	69	26.4
Residence	Urban	131	50.2
	Rural	130	49.8
History of previous Hospitalization	Yes	107	41
	No	154	59
Ward condition	Private	43	16.5
	Public	202	77.4
	Others	16	6.1
Total duration of hospital stay	<7 days	177	67.8
	> = 7 days	84	32.2

### Surgery related factors

Informed consent was obtained from all the participants. The Majority, 62.8% of the participant underwent elective surgery. Sixty-three percent of the participant’s hand no previous history of surgery. Abdominal surgery was conducted among 42.9% of the participants. The total duration of surgery lasted from 1-2hrs among 54.8% of the participants. About half 50.6% of the respondent lost 500-1500ml of blood during the surgery. Only 9.6% of the participants had an implant inserted at the site of operation (Table 2).

**Table 2:** Surgery related factors of the participants (n = 261).

Variables		Frequency	Percentage
Type of surgery	Elective	164	62.8
	Emergency	97	37.2
Previous history of surgery	Yes	96	36.8
	No	165	63.2
Site of operation	Abdominal	112	42.9
	Extremity	49	18.8
	Thorax	24	9.2
	Neck	27	10.3
	Others	49	18.8
Duration of surgery	<1 hr.	49	18.8
	1–2 hr.	143	54.8
	3–4 hr.	43	16.5
	>4hr.	26	10
Amount of blood loss during surgery	<500ml	85	32.6
	500-1500ml	132	50.6
	>1500ml	44	16.9
Implant inserted at site of operation	Yes	25	9.6
	No	236	90.4

### Comorbidities and wound related factors

Among the participants, 20 (7.7%) had a comorbid medical condition and among them, 8 (3.1%) were diabetes mellitus patients. The remaining others had hypertension 6(2.3%), HIV/ADIS 3(1.1%) and Malignancy 3(1.1%). Majority 248(95%) of the participants received wound care as ordered. Among them, 62.8% received twice daily (Table 3).

**Table 3:** Comorbidities and wound related factors of the study participants.

Variables		Frequency	Percentage
Presence of comorbidities	Yes	20	7.7
	No	241	92.3
Types of comorbid (n = 20)	Diabetes mellitus	8	3.1
	Hypertension	6	2.3
	HIV/AIDS	3	1.1
	Ca/Malignancy	3	1.1
Wound care given as ordered	Yes	248	95
	No	13	5
Frequency of wound care (n = 248)	Once daily	60	23
	Two times daily	164	62.8
	Three and more times a day	24	9.2

#### Anesthesia and medication related factor

Majority 66.7% of the study participant received general anesthesia and about half 50.2% of the study subject received the anesthesia for the duration of 30–60 min. Antibiotic prophylaxis was given for 86.6% of the study participants. Ninety-three percent of the participants received medication as ordered (Table 4).

**Table 4:** Anesthesia and medication related factor of the study participants.

Variables		Frequency	Percentage
Type of anesthesia given	General	174	66.7
	Spinal	62	23.8
	Regional	25	9.6
Duration of anesthesia given	<30min	59	22.6
	30-60min	131	50.2
	60-90min	47	18
	>90min	24	9.2
Antibiotic prophylaxis given	Yes	226	86.6
	No	35	13.4
Medication given as ordered	Yes	244	93.5
	No	17	6.5

#### Magnitude of surgical site infection

**Fig 1:**
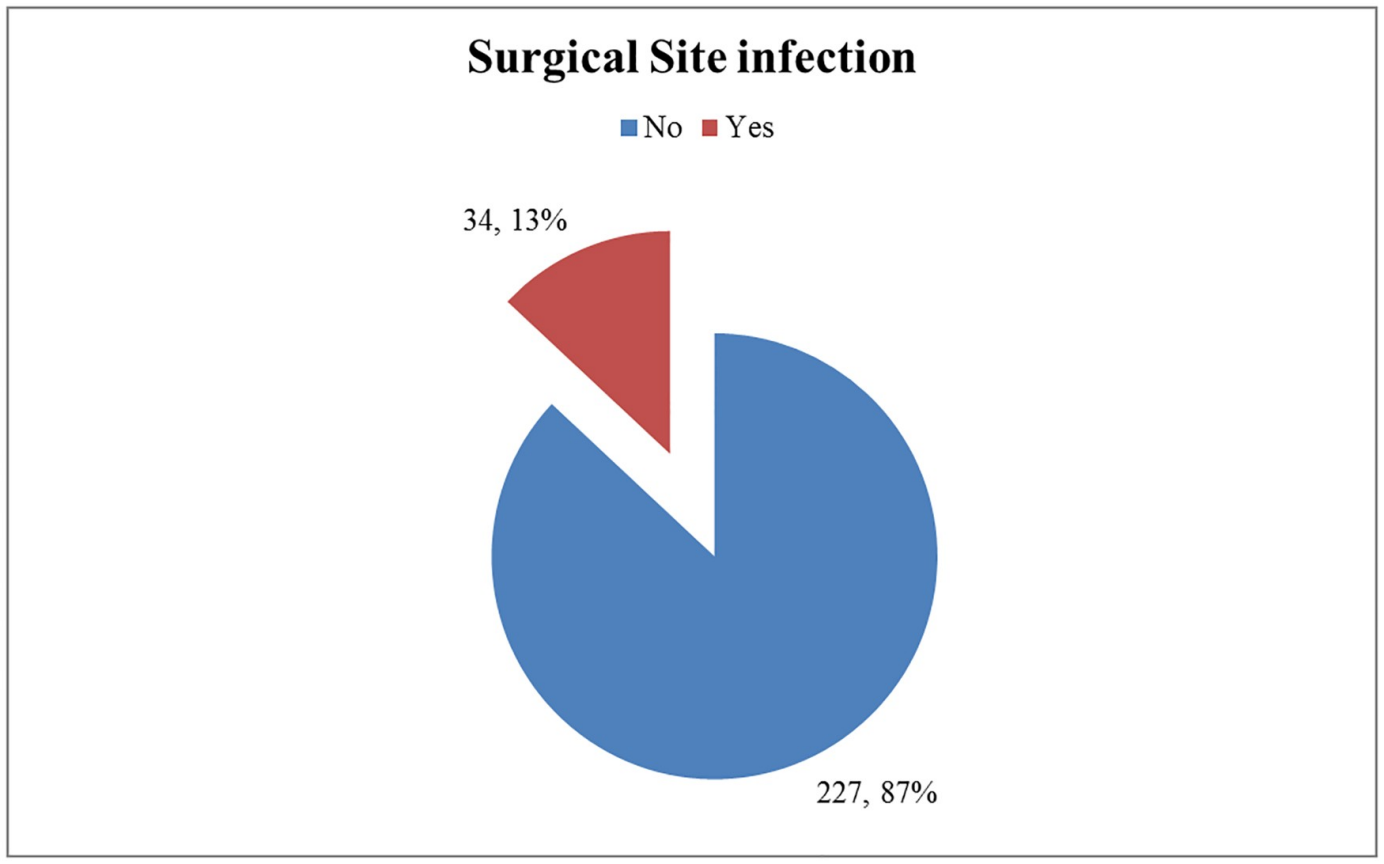
The magnitude of surgical site infection at Wolaita Sodo University Teaching and Referral Hospital.

The magnitude of Surgical Site infection in this study was found to be 13% (95% CI = 9.2%, 17.2%) (Fig 1).

#### Factors associated with surgical site infection

There were 15 variables in binary logistic regression that had a p-value of ≤ 0.25 and became a candidate for multiple logistic regressions. In multiple logistic regressions, only five were significantly associated with surgical site infection, with P value ≤ 0.05. Patients whose age is between >40 years were 6.45 times more likely to develop surgical infection compared to the age group of 1–18 years [AOR 6.45; 95%Cl (1.56, 26.67)]. Illiterates were 4.25 times more likely to develop surgical site infection compared to literate [AOR 4.25; 95%Cl (1.52, 11.84)]. History of the previous hospitalization was significantly associated with surgical site infection; patients who had hospitalization history were 4.5 times more likely to develop a surgical infection than those who had no history [AOR 4.50; 95%Cl (1.44, 14.08)]. Patients who had prolonged preoperative hospital stay (≥7 days) were 3.88 times more likely to develop surgical site infection compared to those who had < 7 days of the stay [AOR 3.88; 95%Cl (1.46, 10.29)]. Patients who admitted on the public wing of the ward were less likely to develop surgical site infection compared to patients admitted on a private wing [AOR 0.24; 95%Cl (0.07, 0.79)] (Table 5).

**Table 5:** Factors associated with surgical site infection.

Independent variables		Frequency	SSI		COR (95% CI)	AOR (95% CI)
			Yes n (%)	No n (%)		
Sex	Female	97	9(9.3)	88(90.7)	1	1
	Male	164	25(15.2)	139(84.8)	1.76(0.78, 3.94)	1.31(0.44, 3.92)
Age	1–18	58	5(8.6)	53(91.4)	1	1
	19–40	81	5(6.2)	76(93.8)	0.70(0.19, 2.53)	2.0(0.36, 11.15)
	>40	122	24(19.7)	98(80.3)	2.60(0.94, 7.20)	6.45(1.56, 26.67)*
Educational status	Literate	155	10(6.5)	145(93.5)	1	1
	Illiterate	106	24(22.6)	82(77.4)	4.24(1.93, 9.31)	4.25(1.52, 11.84)*
Residence	Urban	131	11(25.6)	120(91.6)	1	1
	Rural	130	23(17.7)	107(82.3)	2.35(1.09, 5.04)	1.91(0.72, 5.07)
History of previous Hospitalization	Yes	107	7(6.5)	100(93.5)	1	1
	No	154	27(17.5)	127(82.5)	3.04(1.27, 7.26)	4.50(1.44, 14.08)*
Ward condition	Private	43	11(25.6)	32(74.4)	1	1
	Public	202	17(8.4)	185(91.6)	0.27(0.12, 0.62)	0.24(0.07, 0.79)*
	Others	16	6(37.5)	10(62.5)	1.75(0.51, 5.93)	1.36(0.24, 7.82)
Preoperative hospital stay	<7 days	177	15(8.5)	162(91.5)	1	1
	> = 7 days	84	19(22.6)	65(77.4)	3.16(1.51, 6.59)	3.88(1.46, 10.29)*
Type of surgery	Elective	164	13(7.9)	151(92.1)	1	1
	Emergency	97	21(21.6)	76(78.4)	3.21(1.52, 6.76)	2.51(0.87, 7.24)
Previous history of surgery	Yes	96	18(18.8)	78(81.3)	1	1
	No	165	16(9.7)	149(90.3)	0.47(0.23, 0.96)	0.59(0.21, 1.66)
Duration of surgery	<1 hr.	49	4(8.2)	45(91.8)	1	1
	1–2 hr.	143	15(10.5)	128(89.5)	1.32(0.42, 4.18)	1.36(0.26, 7.06)
	3–4 hr.	43	8(18.6)	35(81.4)	2.57(0.72, 9.24)	3.18(0.45, 22.51)
	>4hr.	26	7(26.9)	19(73.1)	4.15(1.09, 15.84)	1.19(0.16, 9.05)
Amount of blood loss during surgery	<500ml	85	8(9.4)	77(90.6)	1	1
	500-1500ml	132	16(12.1)	116(87.9)	1.33(0.54, 3.25)	1.45(0.43, 4.82)
	>1500ml	44	10(22.7)	34(77.3)	2.83(1.03, 7.80)	2.97(0.66, 13.42)
Implant inserted at site of operation	Yes	25	5(20)	20(80)	1	1
	No	236	29(12.3)	207(87.7)	0.56(0.19, 1.61)	0.60(0.14, 2.62)
Wound care given as ordered	Yes	248	29(12.3)	219(88.3)	1	1
	No	13	5(38.5)	8(61.5)	4.72(1.45, 15.40)	1.72(0.30, 10.05)
Antibiotic prophylaxis given	Yes	226	25(11.1)	201(88.9)	1	1
	No	35	9(25.7)	26(74.3)	2.78(1.17, 6.61)	2.03(0.57, 7.23)
Medication given as ordered	Yes	244	28(11.5)	216(88.5)	1	1
	No	17	6(35.3)	11(64.7)	4.21(1.44, 12.27)	1.98(0.38, 10.21)

* = p-value <0.05,

** = p-value<0.001,

CI = Confidence Interval, COR = Crude Odds Ratio, AOR = Adjusted Odds Ratio SSI = Surgical Site Infection

## Discussion

The Magnitude of Surgical Site Infection in this study was found to be 13% (95% CI = 9.2%, 17.2%). Age, Educational status, Previous history of hospitalization, ward condition, Duration of preoperative hospital stay were factors associated with surgical site infection.

The magnitude of surgical site infection in this study was comparable to study conducted in Ethiopia with the magnitudes of 10.9% in Bahir Dar, North West Ethiopia [13] and 11.1% in Suhul Hospital, Northern Ethiopia [14]. Also study from Saudi Arabia had consistent finding with this study with magnitude 11.4% [15]. But our study was lower than the study conducted in Hawassa with magnitude 19.1%[11] and studies conducted in different parties of Africa with magnitude ranging from (20.6%– 27.56%) [16–18]. But our study was higher than studies conducted in Algeria (5.4%) [14] and Tunisia (8.6%) [19]. This might be attributed to the difference in study design, study period and sample size.

In this study Patients whose age is between >40 years were 6.45 times more likely to develop surgical infection compared to the age group of 1–18 years [AOR 6.45; 95%Cl (1.56, 26.67)] which is consistent with studies conducted in Bahir Dar, North West Ethiopia[13], Hawassa [11], Algeria[14] and Cameroon [18]. This is in fact that as age advances there was an increased incidence of the surgical site. This was also described by different studies in that age is one of non-modifiable risk factor that influence wound healing process and increases the likelihood of a positive surgical outcome [20]. Also in comparison to the younger population, these patients are usually characterized by an impaired immune response to infectious agents, inferior nutritional status, and possibly more comorbidities [21].

The Educational level had a positive effect on surgical site infection. This was also indicated on this study that Illiterates were 4.25 times more likely to develop surgical site infection compared to literate [AOR 4.25; 95%Cl (1.52, 11.84)] this was consistent with the study conducted in Saudi Arabia ^15^. In fact that the levels of educations are important for minimizing perioperative SSI risk through the implementation of recommended process measures [12].

The Previous history of hospitalization was significantly associated with surgical site infection. Indicated in this study patients with the previous history of hospitalization were 4.5 times more likely to develop infection compared to those who had no history [AOR 4.50; 95%Cl (1.44, 14.08)] this was in agreement with a study conducted in India [22]. This might be due to that prior exposure to resistant microorganisms increase the likelihood of the rate of infection [23, 24].

In this study patients who had ≥ 7 days of Preoperative Hospital Stay were 3.88 times more likely to develop surgical site infection compared to those who had less stay [AOR 3.88; 95%Cl (1.46, 10.29)] this is matched with study conducted in India [22], Tunisia [19] and Hawassa [11] this might be due to that global spread of multi-drug resistant infections in health care set-ups and its ubiquitous diagnostic procedures, therapies and microflora have been shown to increase the rate of surgical site infection [23, 24].

### Conclusion

The Magnitude of Surgical Site Infection in this study was high. Age, Educational status, Previous history of hospitalization, ward condition, Duration of preoperative hospital stay were factors associated with surgical site infection. Shortening the preoperative hospital stay, delivery of intravenous antimicrobial prophylaxis before surgery, giving wound care and medication as ordered were important measures to reduce the incidence of surgical site infection.

## Declarations

### Ethics approval

Ethical approval was first got from the Ethical Clearance Committee of Wolaita Sodo University. Then a letter of cooperation written to Wolaita Sodo University Teaching and Referral Hospital (WSUTRH) administration. Participants’ confidentiality of information assured by excluding names and identifiers in the checklist.

### Consent for publication

Not applicable.

### Availability of data and materials

The datasets used and/or analyzed during the current study are available from the corresponding author on reasonable request.

### Competing interests

The authors declare that they have no competing interests.

### Funding

The source of funding for this research was Wolaita Sodo University, College of Health science and medicine. The funders had no role in study design, data collection and analysis, decision to publish, or preparation of the manuscript.
